# Brain activation in persistent postural-perceptual dizziness (PPPD) in response to various visual motion stimuli eliciting abnormal egomotion perception

**DOI:** 10.1016/j.nicl.2026.104068

**Published:** 2026-09-25

**Authors:** Renana Storm, Pia Herborn, Hannah Keller, Skadi Gerkensmeier, Hannah Schewe, Christoph Helmchen, Andreas Sprenger

**Affiliations:** a University of Luebeck, Department of Neurology, Ratzeburger Allee 160, 23538 Lübeck, Germany; b University Hospital Schleswig-Holstein, Campus Luebeck, Ratzeburger Allee 160, 23538 Lübeck, Germany; c Center of Brain, Behavior and Metabolism (CBBM), Marie-Curie-Straße, 23562 Luebeck, Germany; d Institute of Psychology, University of Luebeck, Lübeck, Germany; e The University of British Columbia - Vancouver Campus, 2136 West Mall, Vancouver, BC V6T 1Z4, Canada

**Keywords:** PPPD, Visual stimulation, fMRI, Functional dizziness

## Abstract

**Background:**

Patients with persistent postural-perceptual dizziness (PPPD) perceive unsteadiness associated with abnormal ego- or visual motion perception. We tested for altered functional brain activation of PPPD patients and healthy subjects (HC) while applying various visual motion stimuli (VS).

**Methods:**

Functional brain activation of 32 PPPD patients and 35 age- and gender-matched HC was examined using 3 types of VS-videos: a rollercoaster front row ride (rollercoaster, ROL), optic flow (flowfield, FF), and a silent black-and-white movie (movie, MOV). Fixation of a centered target was used as REST condition. After each trial, the level of visually induced egomotion perception was recorded.

**Results:**

Patients rated VS-induced egomotion larger than HC (ROL > FF > MOV). Brain activation differed between the 3 types of VS for all participants: ROL elicited activation in the Rolandic operculum (OP) and insula, FF in the cerebellum, and MOV in the cingulate cortex and frontal operculum. Whole brain activation for all VS was larger in the patients' visual cortex. Region of interest (ROI) analysis revealed opposite group-related activation patterns in vestibular processing areas: patient's activation was larger during ROL and FF but lower during MOV. Activation in OP2, insula, and uvula became larger with increasing PPPD-handicap and postural sway (FF). VS-evoked brain activation was not correlated with egomotion perception.

**Conclusions:**

The larger activation in the patients' visual cortex during VS supports the notion of abnormal visual dependence, a core feature of PPPD. However, it does not seem to be related to abnormal egomotion perception. Activation patterns of vestibular processing areas suggest that patients differentiate between VS types stronger than HC. VS-related activation in these areas became larger with PPPD-related disability, supporting an abnormal readiness to respond to visual egomotion-inducing stimuli.

## 1 Introduction

Persistent postural-perceptual dizziness (PPPD), one of the established functional neurological disorders (Hallett et al., 2022), is a common chronic neuro-otologic disorder classified by the Bárány society in 2017 (Staab et al., 2017). PPPD is often preceded by peripheral or central vestibular disorders (secondary PPPD) but can also occur without previous acute vestibular events (primary PPPD). Symptoms can be exacerbated by upright posture, active or passive movement, and exposure to moving or complex visual stimuli. Subsequently, a classification of 4 subtypes has been proposed: intolerance to quiet standing or sitting, active motion intolerance, passive motion intolerance, and visual intolerance (Anagnostou et al., 2025). The active motion intolerance subtype appears to be the most prominent subtype in their cohort followed by the visual and quiet standing subtype, irrespective of a previous vestibular disease. However, current diagnostic practice continues to rely on the 2017 Bárány Society criteria as previous studies have identified one to three PPPD subtypes (Park et al., 2024; Yagi et al., 2019).

According to a current pathophysiological model identifying processes that are presumed to precipitate, provoke, and perpetuate PPPD (Staab, 2023; Staab, 2026), patients experience a chronic misperception of (ego-)motion due to excessive body vigilance and lower (ego-)motion perception thresholds. Prior work of our group identified altered perception thresholds for vestibular and visual motion stimuli in PPPD: patients detect egomotion during galvanic vestibular stimulation (GVS) consistently at lower current levels than healthy control subjects (HC) (Helmchen et al., 2024; Storm et al., 2024; Woll et al., 2019). Patients misperceived movement in a quiet sitting position: the lower the threshold of egomotion perception was during passive vestibular chair rotation, the greater the risk of experiencing egomotion without actual chair rotation (Storm et al., 2024). In contrast, the visual motion perception threshold was elevated in PPPD patients: when facing rotating dot kinematograms, patients needed a larger percentage of dots coherently moving in the same direction to correctly identify the direction compared to HC (Storm et al., 2024). Altered sensory perceptional thresholds (Kobel et al., 2023; Storm et al., 2024; Wurthmann et al., 2021) may lead to abnormal egomotion and postural misperception in PPPD which is counterbalanced by lower limb muscle co-contractions leading to a stiffened postural control (Wuehr et al., 2017).

Accordingly, visual dependence as a maladaptive overreliance on visual inputs following a previously experienced vestibular disorder (Cousins et al., 2014) may be related to a shift of both visual and vestibular thresholds. Several studies provided some evidence in favor of a mechanism of reweighting space-motion inputs to prefer visual cues: patients showed a higher intraindividual variability (reduced precision) on estimations of the subjective visual vertical (SVV) during static and on-axis yaw rotation with normal mean data (Jauregui-Renaud et al., 2024). The variability and the magnitude of the deviation of the SVV during concurrent visual motion stimulation is suggested as a parameter for visual dependency in patients with chronic vestibular symptoms (Staab, 2026). However, these findings could not be replicated in two other studies measuring the SVV in different head-tilted conditions in which perceptual errors only occurred in tilted (± 30°/60°) but not in upright (0°) positions (Na et al., 2026; Yagi et al., 2021). Additionally, visual vertical perception seems to be intact as there was no difference in rod deviation between PPPD patients and HC using the Rod-and-Disc test (De Vestel et al., 2022). Conceivably, abnormal perception of verticality while facing rotating dots may impair postural control during visual stimulation. However, data are ambiguous so far, i.e., PPPD patients had increased postural sway compared to HC in one study (De Vestel et al., 2022) while they did not differ from HC in a related study (Storm et al., 2026). In both studies, however, patients indicated larger scores on the visual vertigo analog scale (De Vestel et al., 2022) and larger egomotion perception (Storm et al., 2026) when exposed to visual stimuli (i.e., a rollercoaster ride).

Abnormal reliance on visual cues in PPPD has also been suggested by brain imaging studies. Brain activation in the anterior bank of the central insular sulcus of PPPD patients was lower compared to HC during visual vertical rollercoaster simulation (virtual reality) and decreased with larger activation in patients' visual cortex (V1–3) (Riccelli et al., 2017). The latter relation depended on the dizziness handicap inventory score. This activation difference may be related to the aversive valence of the visual stimulation. However, when exposed to anxiety and dizziness evoking pictures, PPPD patients showed larger activation in vestibular processing areas (retroinsular cortex, superior temporal gyrus) compared to both patients with anxiety disorders and HC (Maywald et al., 2023).

Other studies using various forms of complex visual stimulation by checkerboard and optokinetic stimulus patterns revealed no group activation differences in visual or vestibular processing areas (Yagi et al., 2023), similar to *Popp* et al., who found no abnormal processing in cortical visual, visual motion or vestibular regions during stationary and moving dots visual simulation (7°/s) in patients with phobic postural vertigo, a related disorder preceding the classification of PPPD (Popp et al., 2018).

Taken together, inconsistent results in previous studies do not allow us to deduce a convincing brain mechanism that accounts for visually induced egomotion perception in PPPD patients. The aim of our study was to elucidate brain mechanisms which account for immediate visually-induced egomotion perception by exposing PPPD patients and HC to various visual stimuli, varying in the magnitude of elicited egomotion perception. The specificity of evoked brain activation was designed to be obtained through its relationship with the immediate individual ratings of stimulus evoked egomotion perception, clinical (disease duration, PPPD-handicap) and behavioral parameter (postural sway). We hypothesized (*i*) group differences in the perception of visually-induced egomotion, (*ii*) stimulus-related brain activation differences not only in visual motion, but also vestibular processing areas, and (*iii*) a relation of these differences with behavioral (postural imbalance) and disease-related parameters of functional impairment.

## 2 Methods

### 2.1 Participants

We examined 32 patients (female [f]: 23) and 35 age- and gender-matched (f: 27) healthy control subjects (HC) in this study. Patient recruitment took place at the University Centre for Dizziness and Vertigo in Lübeck, Germany. Only patients who met the diagnostic PPPD criteria of the Bárány Society (see supplementary table **S1**) (Staab et al., 2017) were included. Exclusion criteria included cerebral lesions (i.e., stroke, hemorrhage or trauma), substance abuse including regular sedating medication, severe psychiatric disorders (i.e., depression, anxiety disorder, visual impairment as well as ongoing comorbidities (only HC): migraine, vestibulopathy, benign paroxysmal postural vertigo, tinnitus or acute hearing loss. No participant received antidepressant medication during the study period. All participants received at least nine years of schooling and had no language barrier. All participants filled out seven questionnaires prior to their investigation: the Niigata PPPD Questionnaire (NPQ (Yagi et al., 2019)), the Athens-Luebeck-Questionnaire [ALQ (Anagnostou et al., 2025)] for PPPD-Subtyping, the Hospital Anxiety and Depression Scale (HADS (Hinz and Brahler, 2011; Norton et al., 2013)], the NEO-Five-Factor Inventory [NEO-FFI (Costa Jr and McCrae, 1997)), the short form of the Motion Sickness Susceptibility Questionnaire [MSSQ (Golding, 1998)], the EQ-5D-5L Questionnaire (Hinz et al., 2014) to determine to current quality of life, and the Edinburgh Handiness Questionnaire (Oldfield, 1971). Three HC and four patients were ambidextrous, five patients were left-handed. Apart from a thorough bedside neurologic examination, all participants were investigated using video-based quantitative head impulse test via the EyeSeeCam® HIT System (Autronics, Hamburg, Germany), subjective visual vertical (normal reference: ± 2.5°) as well as ocular vestibular evoked myogenic potentials prior to the study. For further details see (Helmchen et al., 2024; Storm et al., 2026; Storm et al., 2024). Eleven patients had a previous vestibular episode of paroxysmal positional vertigo or vestibular neuritis but none of them or the other participants showed abnormal values at the time of MRI recordings. The study protocol was conducted in accordance with the Declaration of Helsinki and its later amendments and approved by the local Ethics Committee of the University of Lübeck (AZ: 21–098). Written informed consent was obtained from all participants.

### 2.2 Image acquisition

Structural and functional MR imaging was performed at the Center of Brain, Behavior and Metabolism (CBBM) Core Facility for Magnetic Resonance Imaging using a 3-T Siemens Magnetom Skyra scanner equipped with a 64-channel head-coil. Functional images were acquired applying a single-shot, gradient-recalled, echo-planar imaging (GRE-EPI) sequence sensitive to BOLD contrast (TR = 1020 ms; TE = 29 ms; flip angle = 70°; in-plane resolution 3 × 3 mm^2^; 512 × 512 mm^2^ field of view; 60 transversal slices; 3 mm slice thickness; simultaneous multi-slice factor 4); 4 runs of each 551 volumes were recorded. Additionally, structural images of the whole brain using a 3D T1-weighted MP-RAGE sequence were acquired (TR = 1900 ms; TE = 2.44 ms; TI = 900 ms; flip angle 9°; 1 × 1 × 1 mm^3^ resolution; 192 × 256 × 256 mm^3^ field of view; acquisition time 4.5 min). T1-weighted images were inspected for pathological findings by a neuroradiologist. Head movements were minimized using ear pads (Multipad Ear, Pearltec Technology AG, Schlieren [CH]). Ear plugs were used for noise cancellation. During recordings, eye movements were recorded via a video based eyetracker (Eyelink 1000 Plus, 1000 Hz sampling rate, SR Research Ltd., Ottawa, ON/Ca) to monitor eye movements.

### 2.3 Visual stimulation

We introduced participants to three types of visual stimulation (VS) each lasting 12 s, designed to differ by the degree of elicited egomotion: a black-and-white silent movie (movie, MOV), a flow-field animation (flow-field, FF), and a rollercoaster video from the driver's perspective (rollercoaster, ROL, Fig. 1A-C). For movie VS, sections of the movie “The Artist” (Hazanavicius, 2011) were trimmed to 12-s snippets. Flow-field stimulation was programmed using Matlab (version R2022b, The Math-Works, Natick/MA) and the Psychophysics Toolbox 3 extension (version 3.0.16 (Brainard and Vision, 1997), see supplementary data for a video example [FlowField.mp4]). For the rollercoaster VS, sections of publicly available first-row-perspective rollercoaster videos (see Video References in the supplementary data) were trimmed to 12-s snippets. VS were presented on a monitor (NordicNeuroLab LCD Monitor, 32-in. diameter, screen resolution: 1920 × 1080 pixel, 60 Hz refresh rate) 1.34 m behind the participant.

**Fig. 1:**
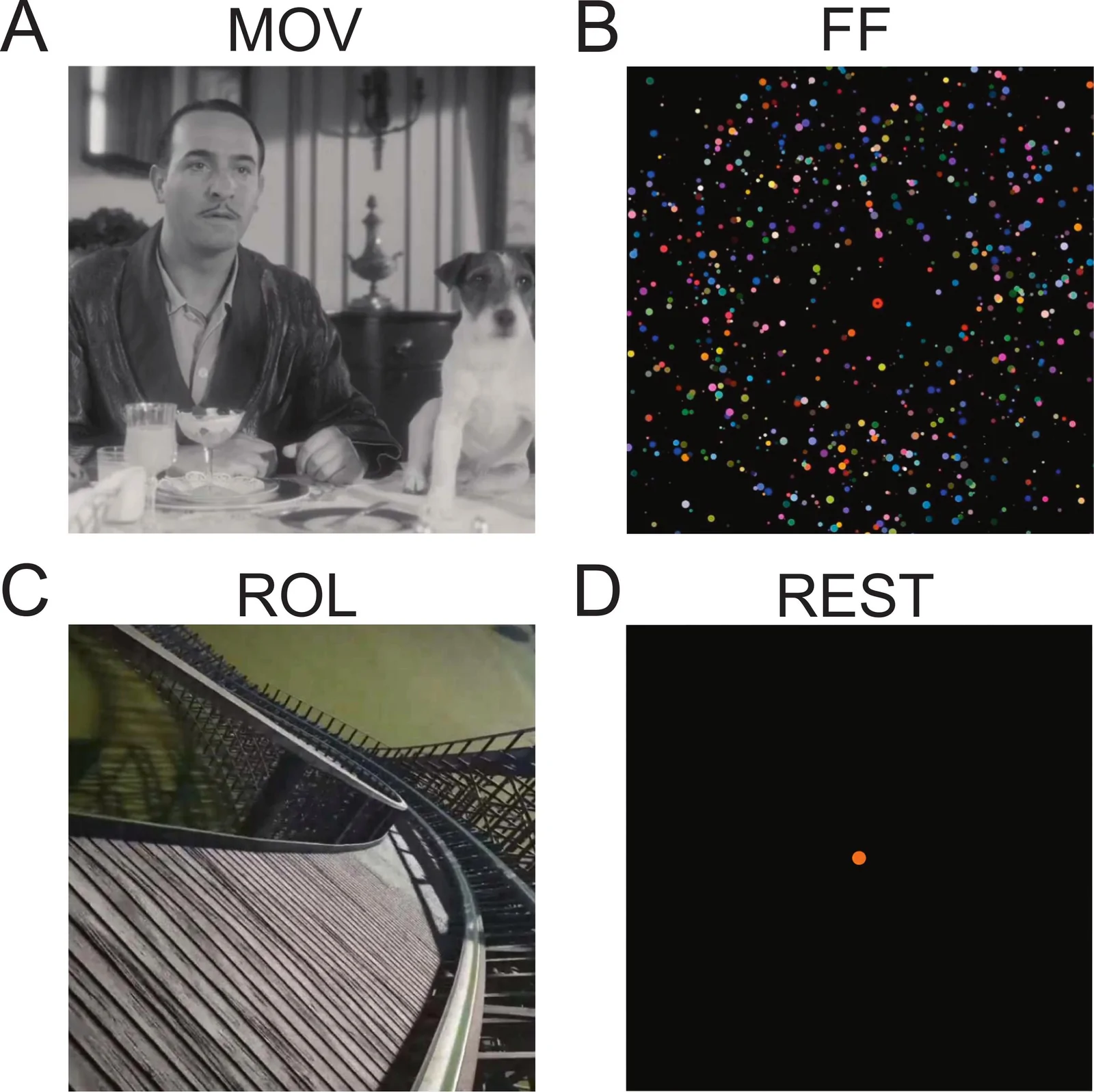
Illustration of the visual stimulation paradigms MOV (A), FF (B), ROL (C). Each stimulus was designed to differ by the degree of elicited egomotion, lasting for 12 s and analyzed against REST (D). Abbreviations: FF = flowfield visual stimulation, MOV = movie visual stimulation, REST = resting condition, ROL = rollercoaster visual stimulation.

### 2.4 Experimental procedure

The total imaging acquisition time was 65 min. Participants were positioned in standard procedure with a specially designed eye tracking mirror placed above their face to see the monitor behind them. The lights were switched off during examination. The T1-weighted image was recorded in the first 5 min. A joystick (Tethyx, HHSC-JOY-5, Current Designs Inc., Philadelphia (PA)) was placed on the right-hand side to manually rate egomotion perception intensity following each visual stimulation during tb-fMRI on a visual analogue-scale from 0 (no) to 100 (most intensive egomotion) presented on the monitor behind them. Participants were carefully instructed to only rate the effect of the visual stimulation without rating other impacts like darkness, sounds or somatosensory input. The experimental prodecure (e.g., looking on a screen via mirror, the use of the joystick) were explained and practiced beforehand. Each VS was replicated two times in pseudorandomized order with a ten-second break in-between. In-between breaks (= resting condition), a central orange dot was presented on the monitor. Including the rating time after each VS (4 s per rating), each run took about 9.2 min (551 s). Overall, 4 runs were completed with a 2-min break between each run.

### 2.5 Posturographic measurements

We correlated brain activation parameters during visual stimulation in patients and HC with their posturographic sway parameters. They were presented the identical 3 visual stimuli outside the scanner while standing on a Kistler force platform (Model 9260AA6, Kistler Instrumente AG, Winterhur Switzerland; 50 cm width, 60 cm length) equipped with piezo-electric 3-component force sensors for recording postural changes. We obtained the postural sway speed (mm/s) as a parameter for postural control. Posturographic data have been published elsewhere (Storm et al., 2026).

### 2.6 Preprocessing and analysis

MRI-preprocessing and first level analysis were performed using the SPM12 software (Wellcome Trust Centre for Neuroimaging, London/UK) implemented in Matlab 2025b (The Mathworks Inc., Natick/MA). Slice timing correction, motion correction by rigid body spatial transformation to the mean functional image of each dataset (individual head motion was <3 mm or 3°, minimum to maximum), spatial normalization to a standard template (Montreal Neurological Institute, MNI), resampling to 3 × 3 × 3 mm^3^ and spatial smoothing (6 mm full width half maximum Gaussian kernel) was completed. We estimated individual head motion by the six realignment parameters, i.e., three rotational and three translational movements with respect to the first image in the EPI series. fMRI time series were modeled using a general linear model (GLM). The GLM included regressors for the start of every type of stimulation trial convolved with the canonical hemodynamic response function of SPM12. The six motion parameters were used as covariates in the GLM. We commenced the following analysis by performing whole brain analysis with a flexible factorial design (Gläscher and Gitelman, 2008) for the factors GROUP (patients, HC) and STIMULUS (movie, flow-field, rollercoaster VS) against resting condition (= REST, Fig. 1D) and the interaction between GROUP and STIMULUS. Next, we contrasted main effects for GROUP and STIMULUS and their interaction against each STIMULUS (e.g., ROL > FF). We applied clusters-wise calculation with limits at a *p*-value <0.05 with family wise error (FWE) correction for multiple testing. Activations were anatomically localized with the Automated Anatomical Labeling (AAL) (Tzourio-Mazoyer et al., 2002), and cytoarchitectonic probability maps (Eickhoff et al., 2007; Eickhoff et al., 2005). Regions of interest (ROI, taken from both brain sides) were defined using the SPM Anatomy Toolbox (Version 3.0 (Eickhoff et al., 2005)). A list of all used ROIs can be found in the supplementary data (supplementary table **S2**).

### 2.7 Statistical analysis

Statistical analyses were performed with SPSS (31.0.1.0; IBM Corp., Somer NY). For comparison of groups and stimulus, we used analysis of variance (ANOVAs) and post hoc *t*-tests which were Bonferroni corrected for multiple testing. Sphericity requirement was violated in several ANOVAs; therefore *p*-values are reported with Greenhouse-Geisser correction. Statistical comparisons were performed parametric unless stated otherwise. Questionnaire results were not normally distributed; therefore, we used the Mann-Whitney-*U* test. For correlation analysis with questionnaires and disease parameters, Spearman-Rho nonparametric correlation was used. Statistical differences were regarded as significant for values *p* < 0.05. Data reported indicate mean values (M) and standard deviation (SD) unless otherwise stated.

## 3 Results

### 3.1 Participants

Separate analysis for primary and secondary cause of PPPD revealed no significant group differences. Therefore, patient data were analyzed as one patient group.

The mean age of the patients was 46.3 ± 13.1 years and 46.7 ± 13.1 years for HC. The mean disease duration was 32 ± 39 months. Static SVV did not differ between groups (patients: 1.07° ± 0.11, HC: 1.03° ± 0.08, *p* = 0.844) whereas dynamic SVV was larger in patients (patients: 1.74° ± 0.166, HC: 1.20° ± 0.11, *p* = 0.014), though within normal range. Patients reported higher NPQ and ALQ scores compared to HC, as expected (NPQ: Z = −7.10, ALQ: Z = −7.13, *p* < 0.001 for both), most prominent in the visual subscores (NPQ visual: Z = −6.70, ALQ visual: Z = −6.70, *p* < 0.001 for both). Patients scored significantly higher values for the HADS (HADS-Anxiety: Z = −5.37; HADS-Depression: Z = −5.95, *p* < 0.001 for both) and for the neuroticism trait of the NEO-FFI (Z = −4.52, *p* < 0.001), whereas the extraversion trait was lower amongst patients (Z = −3.07, *p* = 0.002).

The MSSQ did not differ between groups (*p* = 0.271). The current quality of life was significant lower in patients (Z = −5.68, *p* < 0.001).

### 3.2 Egomotion perception of visual stimulation

There was a main effect for STIMULUS (F(2, 64) = 21.36, *p* < 0.001) and GROUP (F(1, 65) = 6.09, *p* = 0.016) but no interaction GROUP x STIMULATION (*p* = 0.529). As expected, ROL elicited the largest egomotion perception followed by FF and MOV with larger ratings amongst patients in all three conditions (see Table 1 and Fig. 2). Post hoc analysis revealed higher ratings for ROL compared to MOV for both patients (*p* < 0.001) and HC (*p* = 0.005) and to FF (*p* = 0.016) in patients only (HC: *p* = 0.073). Patients (but not HC: *p* = 0.084) indicated larger ratings of perceived egomotion by FF vs. MOV (*p* = 0.006).

**Table 1:** Perceived intensities of egomotion elicited by visual stimulation.

Visual stimulation	PPPD patient scores (%)	HC scores (%)	*p*-value
movie	15.84 ± 3.35	7.38 ± 3.21	**0.024**
flowfield	21.34 ± 3.35	11.02 ± 3.21	**0.025**
rollercoaster	29.04 ± 3.35	16.91 ± 3.21	**0.032**

PPPD patients and HC ratings of the 3 visual stimulations: movie, flowfield and rollercoaster (range 0–100%) with group comparison with corresponding *p*-values (significant *p*-values are marked bold). Abbreviations: HC = healthy control subjects, PPPD = persistent postural-perceptual dizziness.

**Fig. 2:**
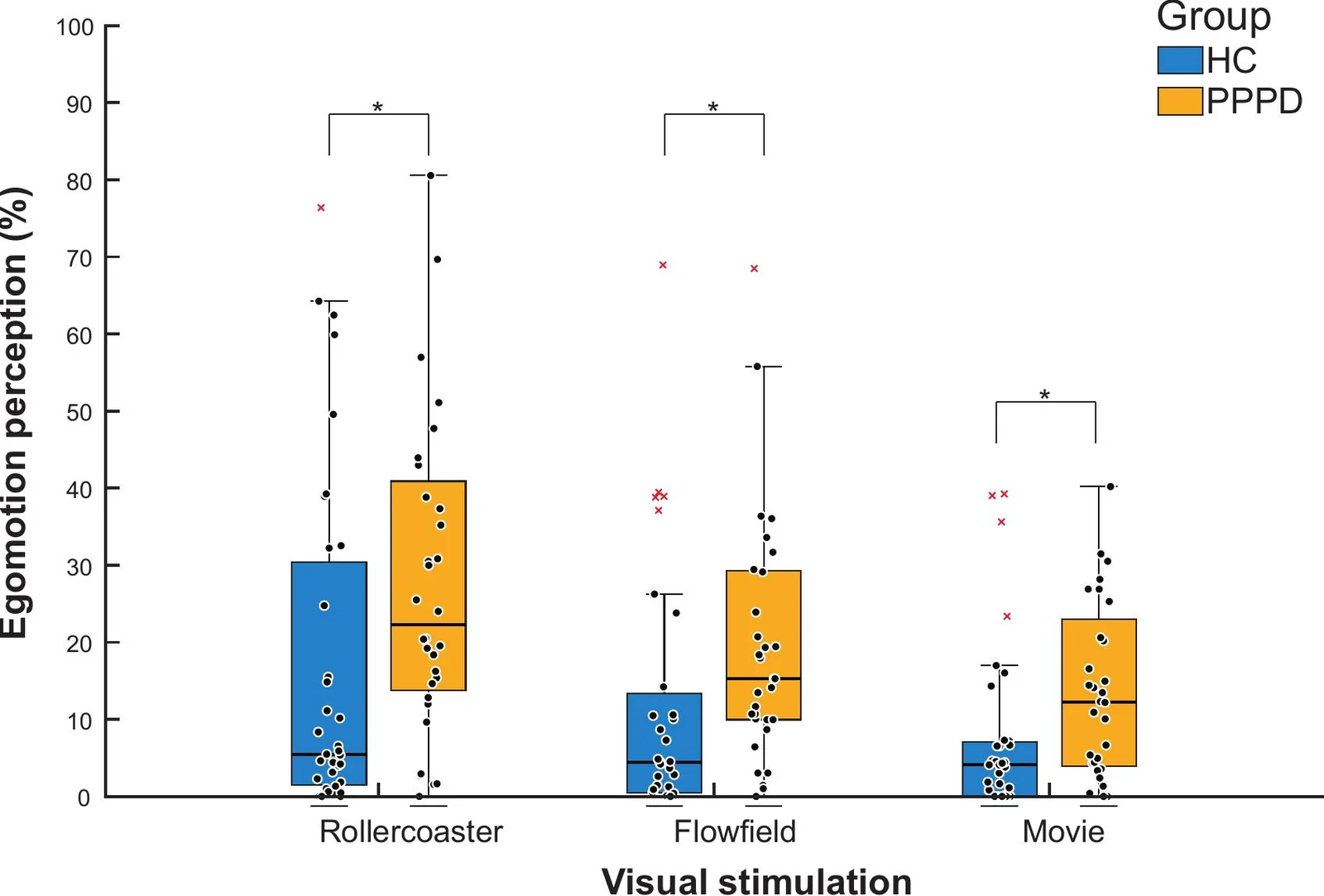
Egomotion perception (%) of each VS compared between groups (HC, PPPD). ROL elicits the highest level of egomotion perception followed by FF with PPPD patients rating each VS higher than HC. * ≤ 0.05. Abbreviations: HC = healthy control subjects, PPPD = persistent postural-perceptual dizziness.

### 3.3 Imaging results

Mean framewise displacement did not differ between PPPD patients (0.286 ± 0.084 mm) and HC (0.251 ± 0.101 mm; t(65) = 1.496, *p* = 0.139).

#### 3.3.1 Stimulus-related brain activation

Brain activation differed between the 3 visual stimuli in patients and HC. All visual conditions activated the calcarine visual cortex. ROL elicited additional activation in brain areas engaged in vestibular processing, i.e., the Rolandic operculum and insula, whereas FF elicited activation in the cerebellum and MOV in cingulate cortex and frontal operculum (FWE, *p* < 0.05, Fig. 3A and B). Contrasting ROL against FF and MOV (Fig. 3C and D), ROL elicited significant brain activation in the inferior parietal lobulus (IPL), inferior frontal operculum (ROL > FF) and calcarine sulcus (ROL > MOV). The contrast FF > MOV showed significant activations in the insula, calcarine sulcus and cerebellum (suppl. **Fig.S1A**). MOV > ROL demonstrated brain activation in semantic temporal brain areas (medial + superior temporal lobe, precuneus, fusiform gyrus, suppl. **Fig.S1B**). Coordinates, cluster size and t-values of stimulus-related brain activation (group independent) are listed in supplementary Table S3.

**Fig. 3:**
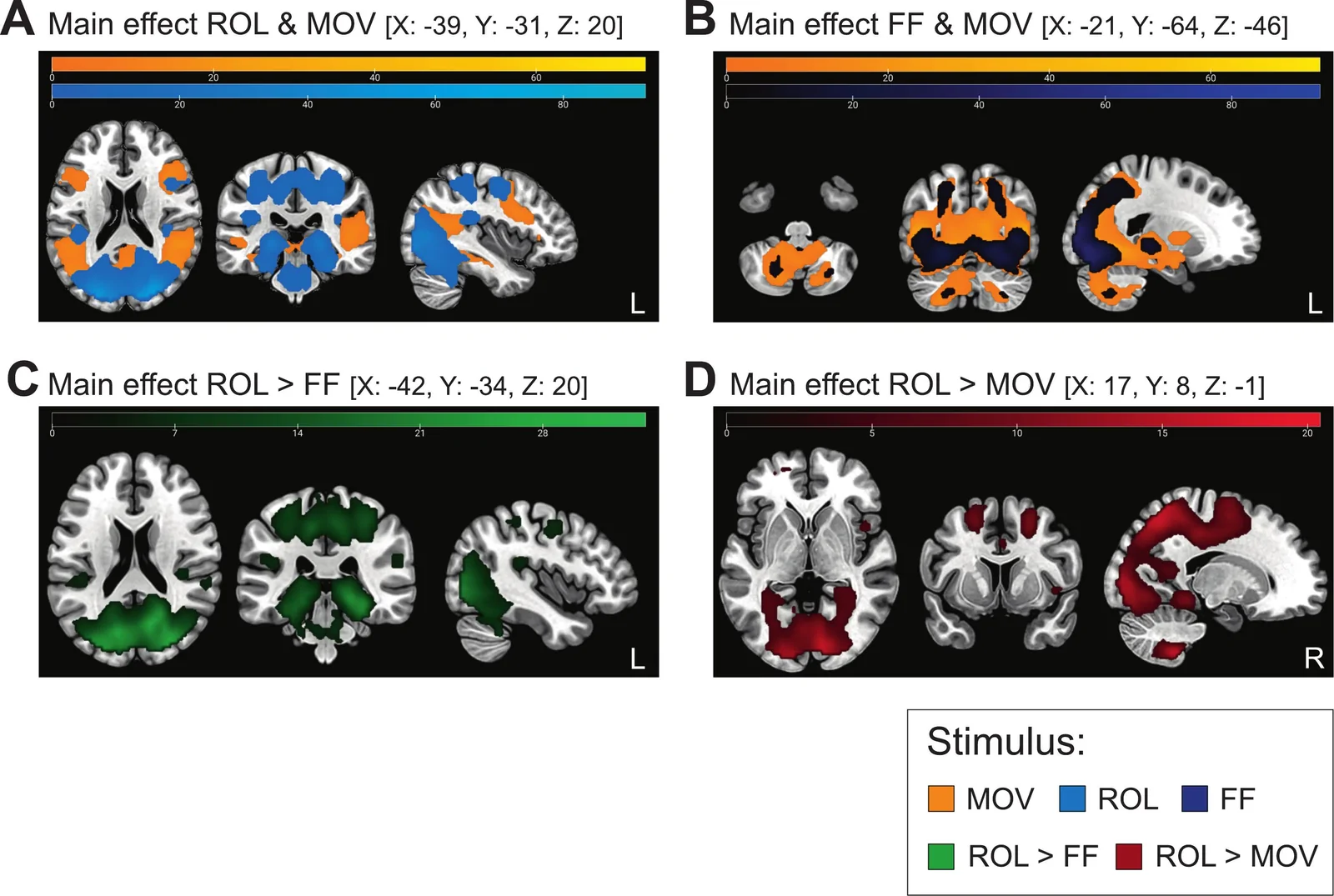
VS-evoked brain activation contrasting ROL (A), FF (B), and MOV (A + B) against REST and visual only contrasts ROL > FF (C) and ROL > MOV (D) (main effect), irrespective of the group (patient/HC). Axial, coronal, and sagittal view (from left to right) for brain areas activated by ROL (blue, rolandic operculum, insula), FF (lilac, cerebellum), and MOV (orange, cingulate and insula cortex) separately] (see also supplementary table S2). The calcarine visual cortex is activated by all three visual conditions. ROL activation only (FF [green] or MOV [red] contrasted) shows a continuous brain activation in vestibular processing areas (operculum, insula) particularly. The coordinates (x, y, z) are given in the Montreal Neurological Institute space. Whole brain analysis was performed using FWE-correction (*p* < 0.05). T-values are indicated along the color bars. Abbreviations: FF = flowfield visual stimulation, HC = healthy control subjects, MOV = movie visual stimulation, REST = resting condition, ROL = rollercoaster visual stimulation, VS = visual stimulation.

#### 3.3.2 Group differences

There was a main effect for GROUP when contrasting brain activation of PPPD patients against HC across all VS (Fig. 4). PPPD demonstrated larger activation in the primary visual cortex (calcarine lobule, lingual, and fusiformis gyrus) which disappeared when analyzing group contrasts for each visual stimulation separately as well as VS contrasts against each other (FWE corrected, *p* < 0.05). Coordinates, cluster size and T-values of group-difference-related brain activation are displayed in supplementary Table S4.

**Fig. 4:**
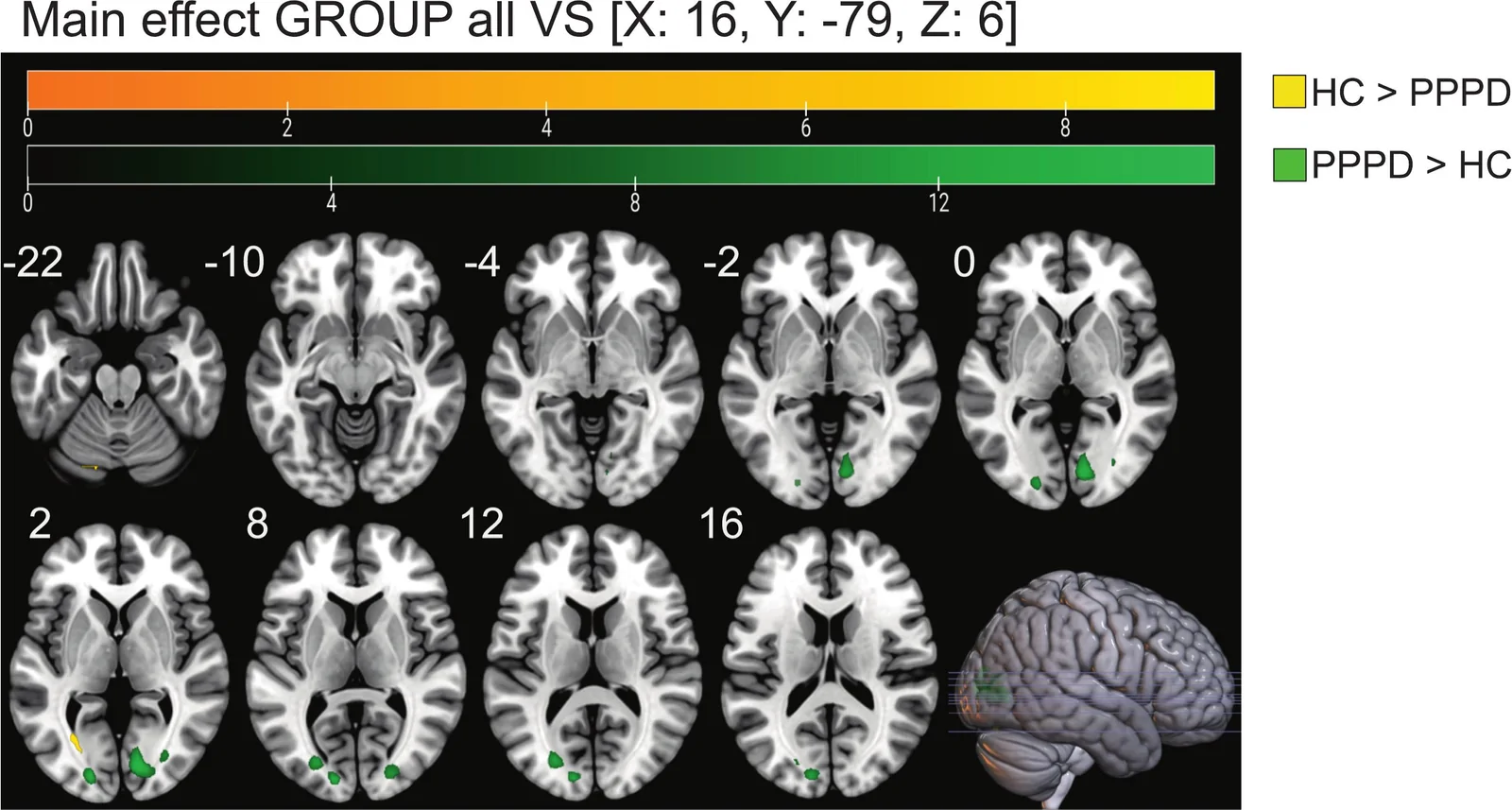
Group-difference related brain activation for all VS against REST: activation of PPPD patients exceeding HC is displayed in green and the reverse contrast (HC exceeding PPPD patients) in yellow. Patients show larger activation in visual processing areas (calcarine sulcus, lingual and fusiform gyrus). Whole brain analysis was performed using FWE-correction (*p* < 0.05). T-values are indicated along the color bars. Abbreviations: HC = healthy control subjects, PPPD = persistent postural-perceptual dizziness, REST = resting condition, VS = visual stimulation.

#### 3.3.3 Region of interest analyses

We performed ROI analyses in visual and vestibular signal processing brain areas as well as brain regions that are known to be involved in human egomotion perception (suppl. Table **S2**) (Ruehl et al., 2022). ROI analysis revealed no significant group differences in brain activation contrasting ROL and FF against REST. However, there was a larger activation in the patients' left primary visual cortex (V1) for ROL > FF (t(65) = −2.18, *p* = 0.033), the right cerebellar lobulus 4/5 for ROL > MOV (t(65) = −2.42, *p* = 0.018) and in the right IPL (t(65) = −2.76, *p* = 0.008) and supramarginal gyrus (SMG) bilaterally for FF > MOV (left: t(65) = −2.67, *p* = 0.009; right: t(65) = −2.39, *p* = 0.020, Fig. 5A-D). Contrasting MOV against REST, patients showed lower activation in the right IPL (t(65) = 2.09, *p* = 0.040) and in the supramarginal gyri bilaterally (left: t(65) = 2.01, *p* = 0.048, right: t(65) = 2.24, *p* = 0.029) (Fig. 5E and F).

**Fig. 5:**
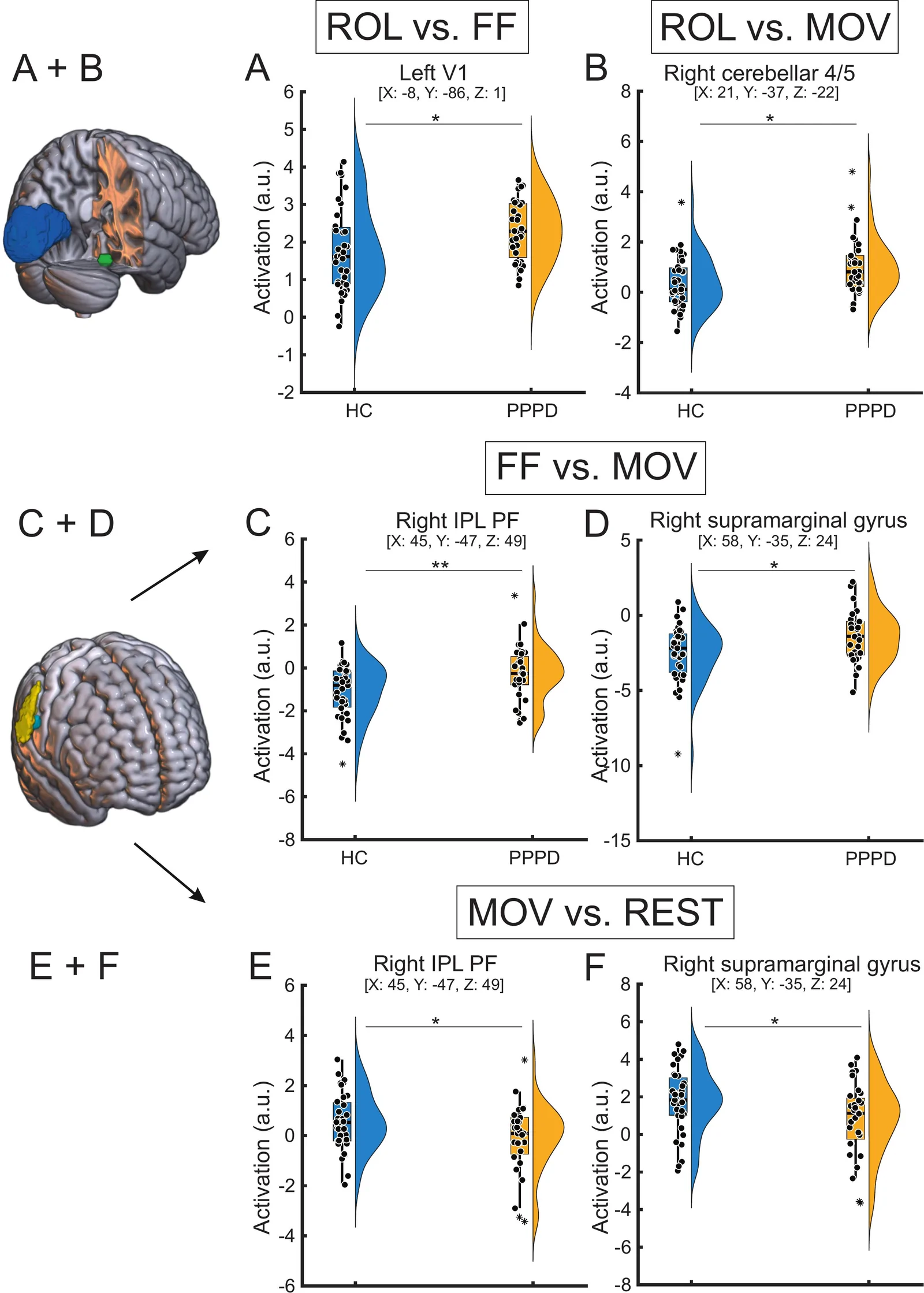
Group comparisons (PPPD vs. HC) for brain activations elicited by VS against another VS (ROL vs. FF (A), ROL vs. MOV (B), FF vs. MOV (C + D)) or against REST (E + F) using ROI analysis. During ROL and FF, patients show larger activation in visual (V1) and vestibular (cerebellum, IPL, supramarginal gyrus) areas compared to HC. MOV, however, revealed lower brain activation in vestibular processing areas in patients. The coordinates (x, y, z) are in the Montreal Neurological Institute space. Statistics: ROI analyses, FWE *p* < 0.05 corrected. * ≤ 0.05, ** ≤ 0.01. Abbreviations: FF = flowfield visual stimulation, HC = healthy control subjects, IPL = inferior parietal lobe, MOV = movie visual stimulation, PPPD = persistent postural-perceptual dizziness, REST = resting condition, ROL = rollercoaster visual stimulation.

#### 3.3.4 Correlation with clinical and behavioral data

Within these ROIs, VS-induced activation in visual and vestibular processing areas (parietal operculum, IPL, insula, V1–5) increased in patients with larger PPPD associated symptom severity (NPQ, ALQ) for ROL, FF but also MOV against REST (Fig. 6A and B). This was even more pronounced in patients with the dominant visual subtype and when ROL activation was contrasted against FF and MOV (see Table 2). Brain activation in the right IPL and bilateral SMG became larger with increasing sway velocities when contrasting FF against REST in upright position, in both patients and HC. (Fig. 6C). Activations in both groups were not correlated with age, individual ratings of egomotion perception, anxiety, depression, or disease duration.

**Fig. 6:**
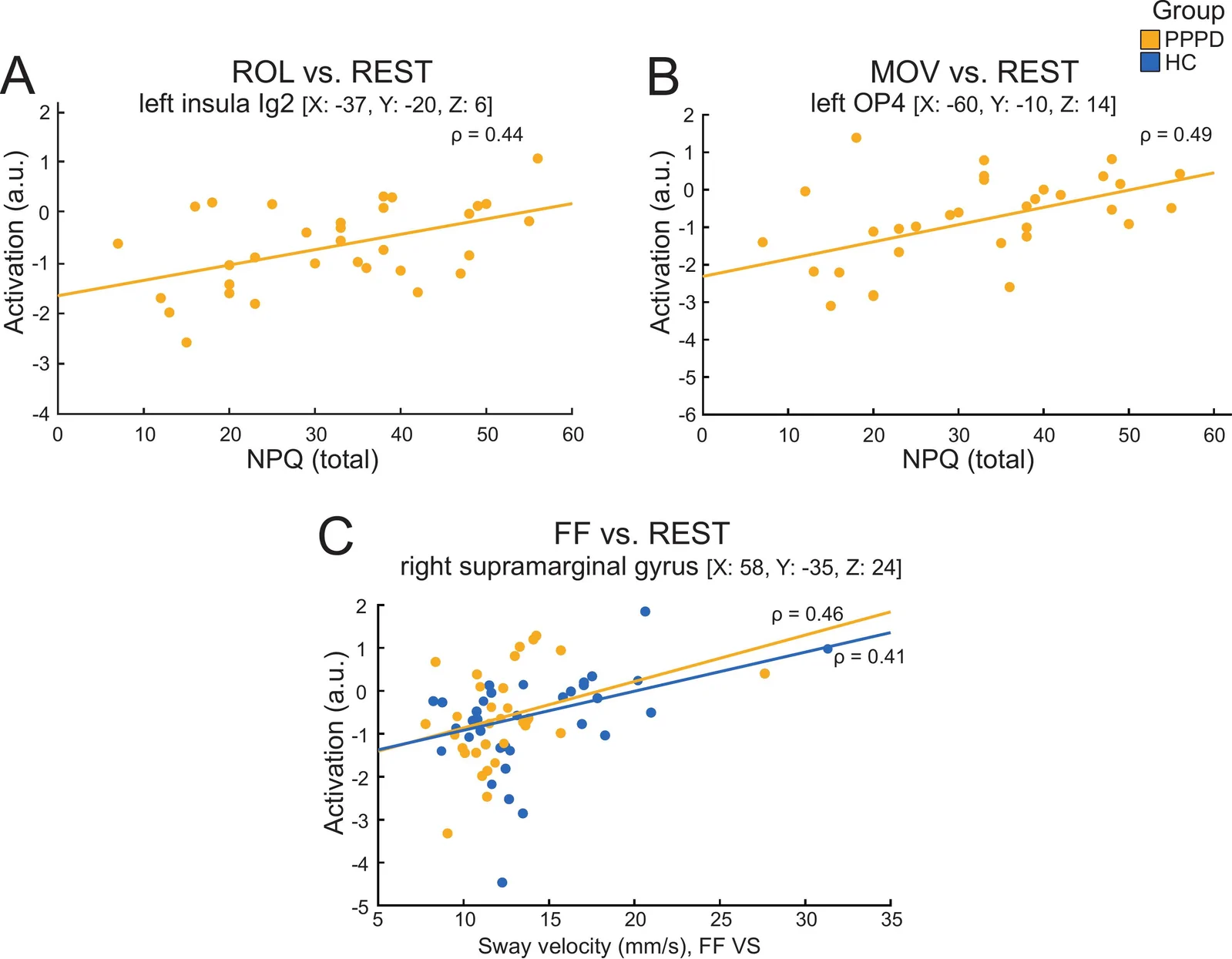
Activation of vestibular processing areas (insula, OP4, supramarginal gyrus) by ROL (A), MOV (B) and FF(C) increases with level of PPPD-related functional disability (NPQ) and postural sway speed during the same VS (FF) on firm surface. Patients are marked orange, HC blue. The coordinates (x, y, z) are given in the Montreal Neurological Institute space. Statistics: Spearman correlation (ρ), *p* always <0.01. Abbreviations: FF = flowfield visual stimulation, HC = healthy control subjects, MOV = movie visual stimulation, MSSQ = Motion Sickness Susceptibility Questionnaire, NPQ = Niigata PPPD Questionnaire, OP = parietal operculum, PPPD = persistent postural-perceptual dizziness, ROL = rollercoaster visual stimulation, VS = visual stimulation.

**Table 2:** Significant correlations of brain activation with clinical and behavioral parameters.

Contrast	Behavior	Brain region	PPPD: corr. (ρ)	PPPD: *p*-value	HC: corr. (ρ)	HC: p-value
ROL > REST	NPQ	insula Ig2 [R]	0.46	0.009	−0.11	0.518
	NPQvis	insula Ig2 [L + R]	0.45 0.47	0.01 0.006	−0.16 −0.29	0.351 0.097
	MSSQ	OP2 [R]	−0.48	0.005	0.36	0.035
	MSSQ	Vermis (uvula) [L]	−0.45	0.009	0.13	0.454
FF > REST	ALQ	V5 [R]	0.47	0.006	0.11	0.52
	MSSQ	OP2 [R]	−0.53	0.002	0.31	0.07
	PSS-FF	IPL [R]	0.50	0.004	0.35	0.042
	PSS-FF	SMG [L + R]	0.47 0.46	0.007 0.008	0.41 0.17	0.014 0.333
MOV > rest	ALQ	Insula Ig2 [L]	0.55	< 0.001	0.17	0.335
	ALQ	OP2 [L]	0.46	0.008	−0.01	0.991
	ALQ	V1 [L]	0.47	0.007	−0.31	0.073
	ALQ	V2 [L]	0.45	0.01	−0.33	0.057
	ALQ	V2 [R]	0.49	0.004	−0.29	0.087
	ALQ	V5 [R]	0.50	0.003	0.01	0.989
	NPQ	insula Ig2 [R]	0.49	0.005	−0.02	0.91
	NPQvis	OP2 [L]	0.46	0.009	−0.23	0.193
	MSSQ	Vermis (uvula) [L]	−0.45	0.01	−0.07	0.681

Abbreviations: ALQ = Athens-Lubeck-Questionnaire, FF = flowfield visual stimulation, MOV = movie visual stimulation, HC = healthy control subjects, IPL = inferior parietal lobe, MSSQ = Motion Sickness Susceptibility Questionnaire, NPQ = Niigata PPPD Questionnaire, OP2 = parietal operculum 2, PPPD = persistent, postural-perceptual dizziness, PSS-FF = postural sway speed during flowfield visual stimulation, ROL = rollercoaster visual stimulation, SMG = supramarginal gyrus, V5 = visual area V5.

## 4 Discussion

Visual dependency is presumed to be a core feature in patients with PPPD. We examined functional brain activation during various visual motion stimulation in relation to perceived visually induced egomotion. We have structured our discussion as follows: (i) discrimination of our visual stimulation paradigms, (ii) visual dependence in PPPD, (iii) visual dependence with regard to perception thresholds, and (iv) sensory-neural amplification.

### 4.1 Discrimination of our visual stimulation paradigms

We hypothesized that brain activation in vestibular and egomotion processing areas would show group differences particularly during ROL accounting for the differences in perceived egomotion intensity of patients during this stimulus. The three paradigms of VS revealed distinctly different levels of egomotion perception in both groups with consistently larger ratings in patients. Exposure to ROL VS elicited the largest levels of perceived egomotion, followed by FF and MOV. This pattern was found for both groups. Furthermore, the three VS conditions evoked different brain activation sites: apart from the visual cortex, ROL and FF activated areas involved in vestibular and egomotion processing (Rolandic operculum, insula, cerebellum) (Ibitoye et al., 2023; Raiser et al., 2020; Ruehl et al., 2022; zu Eulenburg et al., 2012) whereas MOV activated areas involved in the semantic network of visual motion processing (superior and medial temporal lobe, precuneus, fusiform gyrus) (Binder et al., 2009).

### 4.2 Visual dependence in PPPD

Using whole brain analysis, larger bilateral activation in the patients' primary visual cortex (calcarine sulcus, lingual and fusiform gyrus) was identified when all types of VS were analyzed together. This is in line with the suspected visual dependency of dizziness in PPPD patients when patients were exposed to complex visual motion in their visual surrounding (Staab, 2026). However, this group difference did not reach significance for any of the single VS conditions (e.g., ROL only). Thus, our analysis did not succeed in delineating the type of visual stimulation when accounting for the larger visual cortex activation and its relation to the intensity of egomotion perception.

Previous studies using paradigms with a single VS, e.g., vertical rollercoaster, did not identify group differences of activation in the visual cortex, however VS-evoked activation increased with the severity of perceived dizziness handicap (DHI) (Riccelli et al., 2017). The increase of visual cortex activation with the subjective handicap (DHI) has been taken as one neural fingerprint of visual dependence of dizziness in PPPD. Our study yields similar results, by showing larger activation in patients' visual cortex, which also increased with disease related symptom severity (NPQ, ALQ), not only for ROL and FF but also MOV VS. Analysis of all three types of VS revealed greater visual cortex activation compared to HC, suggesting that the robust evoked egomotion perception (in ROL and FF) may account for the group differences, probably due to the use of natural visual motion stimuli.

In another VS study involving patients with the PPPD precursor *phobic postural dizziness*, the patients' visual cortex activation was not different from HC during (black and white dots) and after (motion after effect, MAE) optokinetic stimulation and not correlated with prolonged MAE duration (Popp et al., 2018). One reason for the absence of group differences in the visual cortex may be stimulus-linked as optokinetic stimuli not necessarily induce egomotion due to their stereotypical nature. Yagi and coworkers extended VS by applying different types of static and dynamic VS (checkerboard rows and squares VS at different stimulus frequencies; black-and-white stripes optokinetic stimulation and radial optic flow) (Yagi et al., 2023). Despite the variety of VS, there were no group differences of brain activation, neither in visual nor vestibular processing areas (Yagi et al., 2023). Importantly, however, VS increased functional connectivity to the visual cortex which may account for the visual dependence in PPPD patients. However, perceptional levels of perceived egomotion were not assessed.

In contrast, we used dynamic VS with varying degrees of perceived egomotion which revealed subtle changes of activation in the visual cortex, cerebellum and multimodal vestibular processing areas when performing ROI analysis. As we hypothesized that ROL would elicit the largest level of perceived egomotion, we were interested in delineating the visual motion component that most strongly evoked egomotion perception. Therefore, we subtracted the visual motion component from the ROL VS by contrasting ROL > FF and ROL > MOV or FF > MOV. Previous studies using experimental virtual reality ROL stimuli (Riccelli et al., 2017) or FF (Yagi et al., 2023) paradigms provided no analysis of subtracted contrasts, hindering a direct comparison. Interestingly, with these contrasts, patients showed larger brain activation in cerebellar and multimodal vestibular processing cortical areas (right IPL, right supramarginal gyrus). VS paradigms that strongly evoke egomotion perception more readily activate multimodal vestibular processing areas in patients while non-egomotion evoking VS (e.g., MOV) even shows lower activation in the same areas compared to HC.

### 4.3 Visual dependence with regard to perception thresholds

Neural activation in IPL and SMG seems to depend on visually induced egomotion. Activation of the SMG became larger with increasing posturographic sway speed in both groups using the same VS (FF). This is in line with previous studies identifying visual motion processing in the IPL and SMG additionally to visuospatial integration functions (Pitzalis et al., 2010; Ruehl et al., 2022). Accordingly, SMG activation may be related to egomotion perception of physiological postural sway. This conclusion has to be taken with caution, since VS-related brain activation was not correlated with the immediate ratings of perceived egomotion. In turn, brain activation in insula, OP2, and uvula of the vermis seems to be disease-related, as it became larger with increasing symptom severity (higher scores on the ALQ, NPQ). Severely affected patients seem to have an abnormal readiness of these areas to respond to visual egomotion-inducing stimuli and thereby develop an increased motion sensitivity.

Our three VS paradigms clearly differed in the context-dependent challenge of visual motion detection. Visual coherent motion detection thresholds were found to be elevated rather than lowered (Storm et al., 2024), similar to migraine patients (Antal et al., 2005; Battista et al., 2010). Elevated thresholds, i.e., delayed coherent visual motion detection might lead to anxiety due to later behavioral responses to a delayed recognition of visual motion. MOV stimulation required semantic processing of the movie content but it hardly elicited egomotion perception. Abnormal recognition during this condition is therefore unlikely to influence egomotion perception. Abnormally elevated coherent motion detection thresholds during ROL stimulation, in contrast, would conceivably elicit anxiety due to potentially delayed behavioral responses to ROL evoked egomotion perception (see ROL video). As there were no differences in visual cortex activation between ROL and MOV, we consider it unlikely, that the patients' visual cortex activation is related to anxiety and altered motion detection thresholds. Moreover, SVV was within normal range for all patients in our cohort and the larger activation in the patients' visual cortex was neither correlated with perceived egomotion intensity nor anxiety levels (HADS) in our study.

Conceivably, visual attention and/or anxiety may also contribute to spatial cognition impairment, e.g., in allocentric mapping, with predictive coding errors in updating internal models (Faundez et al., 2025). Spatial anxiety of PPPD patients increases with the larger variability of the subjective visual vertical of PPPD patients, apart from postural sway and perceived dizziness-related handicap (Jauregui-Renaud et al., 2024). Abnormal functional connectivity between anterior insula and the visual cortex (middle occipital cortex) has been related to the larger visual reliance of egomotion perception in the PPPD precursor condition “chronic subjective dizziness” (Indovina et al., 2015). This may also be related to larger levels of anxiety in PPPD which show an overlap with vestibular-sensory networks (Lotze et al., 2025). Therefore, it remains open whether the stronger neural activation to VS reflects altered visual processing, e.g., increased sensitivity due to altered visual motion thresholds or a shift of visual attention and/or anxiety.

### 4.4 Sensory-neural amplification

Likewise, the larger activation of vestibular processing areas in response to egomotion evoking vestibular stimulation (GVS) did not correlate with the immediate ratings of perceived egomotion (Storm et al., 2025). Patients discriminated their egomotion perception on a higher baseline level but proportionally with increasing stimulus intensity. We therefore proposed a sensory-neural amplification, i.e., neural sensitization, as a conceivable pathomechanism rather than an abnormal sensory-perceptual scaling. The same seems to hold for visual motion stimuli in PPPD: VS types differed in the magnitude of elicited egomotion between patients and HC and there were distinctly different activation patterns during the different types of VS. However, neural activity in ROI with significant group differences did not correlate with individual egomotion perception ratings, arguing against an abnormal sensory-perceptual amplification as a mechanism in our PPPD patients. There seems to be a neural amplification of sensory signals without a proportional increase in egomotion perception (sensory-neural amplification or neural sensitization, respectively). This suspected sensitization is in line with the readiness of PPPD patients to perceive egomotion even in no-motion conditions (passive rotating chair) (Storm et al., 2024). Perception of motion in non-motion conditions could be related to anticipation but our visual stimuli were presented in a randomized order and perception ratings were collected at the end of each stimulus.

## 5 Limitations of the study

Since many patients develop PPPD secondary to a previous, often still persisting vestibular disorder, our conclusions cannot be automatically generalized to patients with secondary PPPD. As we found no group difference between patients with primary and the few secondary PPPD, it is likely that our group differences cannot be merely explained by a previous vestibular disorder. This needs to be examined in a larger group of secondary PPPD.

As we mainly focused on eliciting different magnitudes of perceived egomotion, we reduced distracting surroundings, e.g., the sound during a rollercoaster ride as a potential bias. We exclusively performed VS (i.e., ROL and MOV) without concurrent auditory stimuli. This makes our VS paradigms different from real-life conditions but we had to exclude acoustic stimuli potentially affecting egomotion perception and providing an attentional bias.

Postural sway speed and functional imaging were accomplished in different positions (standing vs. supine). Therefore, our conclusion about our correlation analysis between functional brain activation and postural sway speed are limited. However, patients egomotion perception between the different visual conditions was very comparable between the standing and the supine lying position (Gerkensmeier et al., 2026).

Lastly, we cannot determine whether the altered functional brain activation observed in patients with PPPD are disease-specific or merely distinguish patients with PPPD from healthy controls, as we did not include a second disease control group in our study. Therefore, future studies should investigate either another functional neurological or a peripheral vestibular disorder as an additional control group to determine whether the observed alterations are specific to PPPD.

## 6 Conclusion

Dizziness of PPPD patients often increases when exposed to a complex visual surrounding, particularly visual motion. Group differences with larger activation not only in the patients' visual cortex but also vestibular processing areas (IPL, SMG) seem to depend on the egomotion-eliciting component of visual motion stimuli. VS-related activation in vestibular processing areas became larger with PPPD-related functional handicap (NPQ, ALQ) suggesting an abnormal readiness of these areas to respond to visual egomotion-inducing stimuli. PPPD patients seem to develop a higher visual motion sensitivity with a sensory-neural amplification mechanism particularly for VS paradigms eliciting perceived egomotion. Further studies with combined visual-vestibular stimulation are needed to identify how enlarged egomotion-related visual motion sensitivity is changed when exposed to concomitant vestibularly driven egomotion, i.e., head and body movements (vestibular stimulation) during physical rehabilitation of PPPD.

The following are the supplementary data related to this article.

Supplementary Video S1

Supplementary material 1

## CRediT authorship contribution statement

**Renana Storm:** Writing – review & editing, Writing – original draft, Visualization, Methodology, Investigation, Formal analysis, Conceptualization. **Pia Herborn:** Writing – review & editing, Investigation, Formal analysis. **Hannah Keller:** Writing – review & editing, Investigation. **Skadi Gerkensmeier:** Writing – review & editing, Investigation. **Hannah Schewe:** Writing – review & editing, Investigation. **Christoph Helmchen:** Writing – review & editing, Supervision, Project administration, Methodology, Investigation, Conceptualization. **Andreas Sprenger:** Writing – review & editing, Visualization, Supervision, Software, Project administration, Methodology, Investigation, Formal analysis, Conceptualization.

## Ethics approval

The study protocol was approved by the local Ethics Committee of the University of Lübeck (AZ: 21–098) in accordance with the Declaration of Helsinki. Written informed consent was obtained from all participants.

## Ethics declaration

Written informed consent to take part in the study and to publish the article has been obtained from all participants or their legal representatives. The privacy rights of participants have been observed.

This study was performed in compliance with relevant laws, regulatory frameworks and guidelines where the research took place. This study was approved by the University of Luebeck (Approval No. 21–098).

## Funding

This study was supported by a grant of the 10.13039/100005930German Research foundation to C.H. (HE 2689/6-1).

## Declaration of competing interest

The authors declare that they have no competing interests.

## Data Availability

Neurologic-clinical, behavioral (questionnaire, posturography, rating) and fMRI data are not publicly available to preserve individual's privacy. The data are, however, available from the authors upon reasonable request.
