# Supplementary material for: Brain activation in persistent postural-perceptual dizziness (PPPD) in response to various visual motion stimuli eliciting abnormal egomotion perception
Source: Neuroimage Clin. 2026 Sep 25;52:104068. doi: 10.1016/j.nicl.2026.104068 (PMC13654570; doi:10.1016/j.nicl.2026.104068)
Supplement: Supplementary file 1 [file mmc2.docx]

# Supplementary Data

# Methods

**Table 1:** Diagnostic criteria for persistent postural-perceptual dizziness (PPPD)

| **Criterion** | **Description** |
| --- | --- |
| **A. Persistent symptoms** | One or more symptoms of **dizziness, unsteadiness, or non-spinning vertigo** are present on most days for **≥3 months**. Symptoms persist for prolonged periods (typically hours), may fluctuate in severity, and need not be present continuously throughout the day. |
| **B. Exacerbating factors** | Symptoms occur without specific provocation but are exacerbated by **(1) upright posture, (2) active or passive motion regardless of direction or position, and (3) exposure to moving visual stimuli or complex visual patterns**. |
| **C. Precipitating condition** | Symptoms are precipitated by a condition causing vertigo, unsteadiness, dizziness, or impaired balance, including **acute, episodic, or chronic vestibular disorders; other neurological or medical illnesses; or psychological distress**. |
| **D. Functional impact** | Symptoms cause **clinically significant distress or functional impairment**. |
| **E. Exclusion of alternative diagnosis** | Symptoms are **not better accounted for by another disease or disorder**. PPPD may coexist with other vestibular, neurological, medical, or psychiatric conditions. |

*Note: All five criteria (A–E) must be fulfilled for a diagnosis of PPPD. Diagnostic assessment is primarily clinical; there are no pathognomonic findings on physical examination, laboratory testing, or neuroimaging.*

### Visual stimulation: video references

Hazanavicius, M. (Director). (2011). The Artist [Film]. La Petite Reine**;** Studio 37**;** La Classe Américaine**;** JD Prod**;** France 3 Cinéma**;** Jouror Productions**;** uFilm**.**

4K AWESOME Twister Roller Coaster Front Seat POV Knoebels Amusement Park.: <https://www.youtube.com/watch?v=oAJLKDMihnU>

Tremors Front Row POV | Silverwood Theme Park 2022: <https://www.youtube.com/watch?v=zgU8bQDpn-I>

Nitro front seat on-ride 4K POV @60fps Six Flags Great Adventure**.:** <https://www.youtube.com/watch?v=ZmMLSUbRuoM>

**Table 2**: used regions of interest (ROI)

| ROI label | Brain region | XYZ (MNI, left/right) |
| --- | --- | --- |
| cerebellum 4 5 R | right cerebellar lobulus 4/5 | [21, -37, -22] |
| cerebellum VIIa crus I HEM | cerebellar lobulus VIIa crus I hemisphere | [-45, -72, -36]  [36, -75, -36] |
| cerebellum VIIb | cerebellar lobulus VIIb | [-37, -60, -51] |
| CSv | calcarine sulcus (visual) | [-10, -16, 45]  [14, -29, 47] |
| vermis uvula | uvula of the vermis | [0, -55, -20] |
| vermis VII | vermis VII | [-3, -67, -28] |
| hOc1 (V1) | primary visual cortex V1 | [-8, -86, 1]  [12, -82, 3] |
| hOc2 (V2) | primary visual cortex V2 | [-10, -96, 13]  [13, -87, 5] |
| hOc5 (V5) | primary visual cortex V5 | [-43, -75, 1]  [48, -65, 4] |
| supramarginal | supramarginal gyrus | [-58, -38, 42]  [58, -35, 24] |
| OP1 | parietal operculum 1 | [-51, -23, 19]  [51, -19, 18] |
| OP2 | parietal operculum 2 | [-36, -24, 19]  [36, -21, 19] |
| OP4 | parietal operculum 4 | [-60, -11, 14]  [61, -7, 14] |
| insula OP3 | insular/parietal opercular cortex | [-36, 0, 10]  [41, -8, 17] |
| IPL PFcm | inferior parietal lobe | [-51, -37, 23]  [51, -29, 25] |
| IPL PF | inferior parietal lobe | [-61, -36, 38]  [64, -31, 32] |
| Insula Id1 | dysgranular insula cortex | [-40, -19, -5]  [40, -17, -4] |
| Insula Ig1 | granular insular cortex | [-37, -25, 9]  [34, -23, 10] |
| Insula Ig2 | granular insular cortex | [-37, -19, 5]  [37, -17, 6] |
| gyrus praecentralis area 4a | precentral gyrus area 4a | [-7, -26, 67]  [22, -23, 62] |
| gyrus praecentralis area 4p | precentral gyrus area 4p | [-37, -19, 50]  [37, -19, 47] |

The coordinates (x, y, z) are in the Montreal Neurological Institute space.

# Results

### Imaging results – stimulus related brain activity

**Table 3:** Whole brain activation by visual stimulation group independent

| Contrast | Cluster | Brain region | Cluster size | x | y | z | t-value |
| --- | --- | --- | --- | --- | --- | --- | --- |
| ROL > REST | Cluster 1 | left Rolandic operculum | 80 | -39 | -31 | 20 | 11.09 |
|  | Cluster 2 | right insula | 27 | 39 | -28 | -20 | 8.05 |
| FF > REST | Cluster 3 | left cerebellar lobulus 8 | 31 | -21 | -64 | -46 | 6.53 |
|  | Cluster 4 | right cerebellar lobulus 8 | 16 | 30 | -67 | -49 | 5.89 |
| MOV > REST | Cluster 5 | right inferior frontal operculum | 950 | 39 | 14 | 26 | 18.52 |
|  | Cluster 6 | left mid cingulate cortex | 11 | -12 | -19 | 44 | 6.11 |
|  | Cluster 7 | left Insula | 21 | -30 | 26 | 2 | 5.88 |
| ROL > FF | Cluster 8 | left Rolandic operculum | 49 | -42 | -34 | 20 | 7.22 |
|  | Cluster 9 | right insula | 22 | 39 | -28 | 20 | 6.81 |
|  | Cluster 10 | left inferior parietal lobe | 25 | -48 | -22 | 38 | 5.97 |
|  | Cluster 11 | right inferior frontal operculum | 10 | 54 | 17 | 32 | 5.10 |
| ROL > MOV | Cluster 12 | right insula | 19 | 45 | 8 | -1 | 5.43 |
|  | Cluster 13 | right postcentral gyrus | 15 | 57 | -22 | 38 | 5.16 |
|  | Cluster 14 | left calcarine sulcus | 11 | -18 | -58 | 14 | 5.04 |
| FF > MOV | Cluster 15 | right calcarine sulcus | 281 | 12 | -88 | 2 | 17.86 |
|  | Cluster 16 | right cerebellar lobulus 7b | 62 | 39 | -61 | -46 | 7.81 |
|  | Cluster 17 | right insula | 130 | 42 | -10 | -4 | 7.46 |
|  | Cluster 18 | left cerebellar crus 2 | 21 | -39 | -61 | -43 | 5.81 |
| MOV > ROL | Cluster 19 | right superior temporal lobe | 1942 | 48 | -40 | 11 | 19.61 |
|  | Cluster 20 | left medial temporal lobe | 1469 | -48 | -52 | 14 | 15.43 |
|  | Cluster 21 | left fusiform gyrus | 187 | -42 | -52 | -19 | 17.91 |
|  | Cluster 22 | right precuneus | 616 | 3 | -49 | 38 | 14.22 |

Contrasts are either calculated against rest condition or against another visual stimulus (FWE corrected, p < 0.05). Abbreviations: FF = flowfield visual stimulation, MOV = movie visual stimulation, ROL = rollercoaster visual stimulation.

### Imaging results – group differences

**Table 4:** Group contrast of whole brain activation (contrasting visual stimulation vs. rest) of PPPD exceeding HC and HC exceeding PPPD

| Contrast | Cluster | Brain region | Cluster size | x | y | z | t-value |
| --- | --- | --- | --- | --- | --- | --- | --- |
| PPPD > HC | Cluster 1 | right calcarine sulcus | 228 | 12 | -88 | 2 | 15.65 |
|  | Cluster 2 | Left lingual gyrus | 19 | -9 | -79 | -10 | 8.48 |
|  | Cluster 3 | left fusiform gyrus | 15 | -27 | -64 | -13 | 7.80 |
|  | Cluster 4 | right fusiform gyrus | 30 | 30 | -61 | -16 | 6.90 |
| HC > PPPD | Cluster 5 | left cerebellar lobulus 6 | 58 | -15 | -82 | -19 | 9.43 |
|  | Cluster 6 | right lingual gyrus | 23 | 12 | -76 | -13 | 9.37 |
|  | Cluster 7 | vermal lobulus 4/5 | 52 | 0 | -55 | 5 | 7.04 |
|  | Cluster 8 | right thalamus | 28 | 18 | -28 | 8 | 6.16 |
|  | Cluster 9 | right lingual gyrus | 23 | 12 | -76 | -13 | 9.37 |

Elicited brain activation for group contrasts (PPPD exceeding HC or inversely) across all visual stimulations (FWE corrected, p < 0.05). Abbreviations: HC = healthy control subjects, PPPD = persistent postural-perceptual dizziness.

**Supplementary Figure 1:** VS-evoked brain activation contrasting FF > MOV (**A**) and MOV > ROL (**B**) (main effect), irrespective of the group (patients/HC). Axial, coronal, and sagittal view (from left to right) for brain areas more activated by FF compared to MOV (red, insula, cerebellum) and by MOV compared to ROL (orange, temporal lobe, fusiform gyrus, precuneus). FF elicits increased brain activation in vestibular processing areas, whereas MOV leads to increased activation in semantic processing areas. The coordinates (x, y, z) are in the Montreal Neurological Institute space. Whole brain analysis was performed using FWE-correction (p < 0.05). T-values are indicated along the color bars. Abbreviations: FF = flowfield visual stimulation, HC = healthy control subjects, MOV = movie visual stimulation, ROL =rollercoaster visual stimulation, VS = visual stimulation.
